# Physiological and Molecular Response of *Prorocentrum minimum* to Tannic Acid: An Experimental Study to Evaluate the Feasibility of Using Tannic Acid in Controling the Red Tide in a Eutrophic Coastal Water

**DOI:** 10.3390/ijerph13050503

**Published:** 2016-05-14

**Authors:** Byungkwan Jeong, Eui-Suk Jeong, Jacqueline Martha Malazarte, Yongsik Sin

**Affiliations:** 1 Department of Environmental Engineering & Biotechnology, Mokpo National Maritime University, Mokpo 530-729, Korea; bkjeong@knps.or.kr (B.J.); jacquem19@gmail.com (J.M.M.); 2 Laboratory Oil Pollution Research Center, National Park Research Institute, Taean 357-914, Korea; 3 Laboratory Animal Center, Daegu-Gyeongbuk Medical Innovation Foundation, Daegu 701-310, Korea; euisuklove@hanmail.net

**Keywords:** harmful algal bloom, *Prorocentrum minimum*, red tide, tannic acid

## Abstract

Bioassay and gene expression experiments were conducted in order to evaluate the growth and physiology of *Prorocentrum minimum* isolated from a eutrophic coastal water in response to tannic acid. In the bioassay experiments, variations in abundance, chlorophyll (chl) *a* concentration, maximum fluorescence (*in vivo* Fm), and photosynthetic efficiency (Fv/Fm) were measured over the course of a seven-day incubation. Moreover, stress-related gene expression in both the control and an experimental (2.5 ppm TA treatment) group was observed for 24 h and 48 h. The molecular markers used in this study were the heat shock proteins (*Hsp*70 and *Hsp*90) and cyclophilin (*CYP*). The findings show that *P. minimum* can thrive and grow at low concentrations (<2.5 ppm) of tannic acid, and, above this concentration, cells begin to slow down development. In addition, TA concentration of 10 ppm halted photosynthetic activity. At the molecular level, treatment with tannic acid increased the expression of *Hsp*70, *Hsp*90, and *CYP*, and heat shock proteins are more upregulated than the cyclophilin gene. Exposure to tannic acid increased the expression of stress factors over time (48 h) by 10- to 27-fold the expression level of the control group. These results suggest that tannic acid can be used to control harmful algal blooms such as those containing *P. minimum* in eutrophic coastal waters.

## 1. Introduction

Harmful algal blooms (HABs) pose a serious threat to fisheries, public health, tourism, and coastal ecosystems on a pan-global scale [1,2,3,4]. The 1971 red tides off the west coast of Florida in the U.S. caused an estimated economic loss of $20 million, and the toxins released by HABs off the U.S. west coast have taken a heavy toll on fisheries in that area [5,6]. Shellfish-poisoning red tides have damaged many oyster farms in Hiroshima Bay, Japan, resulting in enormous economic losses [7,8]. Because of the frequent occurrence of red tides, South Korean fisheries have suffered considerable revenue losses every year since the 1990s ($60 million in 1995; $18.6 million in 2003) [9].

Recently, industrial development and economic growth have accelerated the rate of population concentration increases in coastal areas, with a particular increase in human preference for waterfront residences. This increasing urbanization of near-shore and coastal areas can aggravate the eutrophication of coastal waters. Such changes to coastal environments, along with the rise in water temperature and prolonged summers caused by global warming, all contribute to the formation of conditions favorable to the growth of harmful algae [10]. As a result, the frequency of red tide events and the damage associated with them is expected to continue increasing. 

Among the harmful algal species, *Prorocentrum minimum* is known to occur in coastal waters around the world, including those of the North American east and west coasts, northern Europe, the Mediterranean, East Asia, and Oceania [11]. In Korea, *P. minimum* with cell densities of up to 36,000 cells mL^−1^ has been reported to occur in the summer months [12]. *Prorocentrum minimum* blooms, which commonly occur in the summer in low-salinity waters and under eutrophic conditions [13], release toxins harmful to both fish and shellfish species, and, in turn, render the fish and shellfish toxic to humans [13,14]. In addition, they have negative effects on various marine organisms, such as submerged aquatic vegetation and benthic fauna [15,16]. In the US, Chesapeake Bay has frequently been plagued by *P. minimum* blooms between the months of April and May, causing severe economic losses to commercial fisheries [17,18,19]. The northern part of the Philippines has also experienced massive fish die-offs that were linked to the toxins released by *P. minimum* bloom, which led to economic losses estimated at $120,000 [20]. The toxic compound produced by *Prorocentrum minimum* has not yet been identified [21], although it was reported that *P. minimum* exhibited neurotoxic effects [22].

HAB mitigation and control technologies are being designed in order to enable the removal or population reduction of red tide-forming organisms, including *P. minimum*. The clay flocculation HAB mitigation technology that is primarily used in Korea, China, and Japan [23,24,25,26,27] is likely to have negative side-effects on the coastal ecosystem in which it is used [28,29,30]. Therefore, development of effective HAB mitigation technology has been recognized as an urgent issue. 

Tannic acid (TA) is a widely known polyphenolic compound synthesized by common plants, such as persimmon (*Diospyros* spp.), chestnut (*Castanea* spp.), oak (*Quercus* spp.), and tea (*Camellia sinensis*) as well as by aquatic plants like *Myriophyllum* spp. [31,32,33], *Ascophyllum nodosum* [34], and *Polygonum limbatum* Meisn. [35]. The exudation of tannic acid and other phenols by the aquatic macrophytes are documented to suppress the abundance of phytoplankton [allelopathic effect] [36]. It has also been experimentally verified that TA inhibits bacterial growth and activity [37,38]. Such studies investigating the effects of TA on microalgae are limited to a few species of cyanobacteria (blue-green algae), diatoms, and green algae [39]. To date, no research results are available regarding the effect of TA on red tide-causing dinoflagellates such as *Prorocentrum minimum.*

While phytoplankton’s physiological response to specific materials is usually estimated via bioassays analyzing abundance, biomass, and growth rate [40,41,42], recent advances in molecular biotechnology (biology and genetics) have led to an increase in the use of stress-related genes in the molecular analysis of planktonic stress response [43,44,45,46,47]. In light of these changes, this study aims to evaluate the feasibility of using TA in red tide mitigation technology, by investigating the response of *P. minimum* in eutrophic coastal water to TA through a bioassay experiment and stress-related gene expression (heat shock proteins (*Hsps*), cyclophilin (*CYP*)) analysis. 

## 2. Materials and Methods 

### 2.1. Sample Collection and Culture

**Figure 1:**
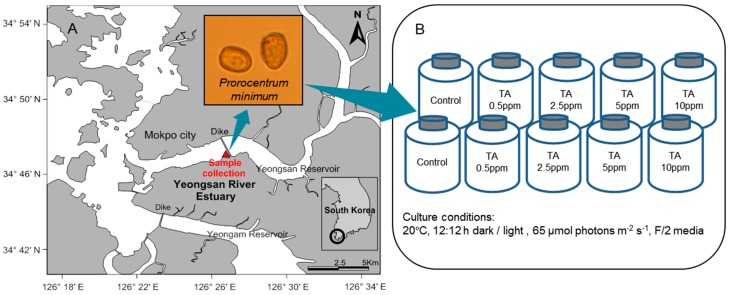
(**A**) location of the collection and isolation of *P. minimum*. (**B**) culture conditions and experimental design to examine the response of *P. minimum* to the various tannic acid (TA) concentrations.

*Prorocentrum minimum* was isolated from a surface layer sample of the Yeongsan River estuary, located in the southwest coast of Korea (Figure 1A) using the capillary method. The Yeongsan River estuary has been eutrophic since a sea dike was constructed 7 km from its mouth [48]. Harmful algal blooms dominated by dinoflagellates such as *Heterocapsa* sp. and *P. minimum* develop during summer especially after eutrophic freshwater discharge from the gates of sea dike [49]. For the tannic acid, commercial TA (CAS No. 1401-55-4) was purchased from Samchun Pure Chemical Co., Ltd. (Gyeonggi-do, South Korea). The set-up was divided into a control group and four experimental groups (0.5, 2.5, 5 and 10 ppm tannic acid concentration) each with equal population densities of *P. minimum* and allowed to grow up to the logarithmic phase (Figure 1B). Cultures were performed in an F/2 media with the following growth conditions: an incubation temperature of 20 °C, 12:12 h dark/light cycle, and an irradiance level of 65 μmol photons m^−2^·s^−1^. 

### 2.2. Bioassay Experiment

During the incubation period, cell abundance, biomass (measured as chl *a* concentration), maximum fluorescence (*in vivo* Fm), and photosynthetic efficiency (Fv/Fm) were measured, in order to quantify the response of *P. minimum* to TA. All measurements were taken in duplicate.

To measure biomass via chl *a* concentration, 10 mL samples were first filtered through glass microfiber filters (Whatman^®^ 25 mm GF/F, Buckinghamshire, England) with a 0.7 µm nominal pore size under ≤100 mm Hg of pressure. Chl *a* was extracted from the filters by putting them into 8 mL light-proof test tubes containing a solution of 90% acetone and 10% deionized water before storing them at 4 °C for 12 h. The chl *a* concentration of the extract (biomass) was then measured using a 10 AU fluorometer (Turner Designs^®^, Sunnyvale, CA, USA). 

Cell abundance was counted by dropping 1 mL of each sample into a Sedgewick–Rafter counting chamber (50 × 20 × 1 mm) and using a ZEISS^®^ Axioskop 2 MAT microscope (Thornwood, NY, USA) for analysis. In order to verify the morphological changes in *P. minimum* exposed to TA, specimens were photographed at 24 h and 48 h after TA exposure, using a Canon^®^ EOS 700D camera (Tokyo, Japan) mounted on the microscope.

Maximum fluorescence (*in vivo* Fm) and photosynthetic efficiency (Fv/Fm) were measured using a FASTtracka Fast Repetition Rate fluorometer (FRRf) (Chelsea^®^ Technologies Group, Surrey, England).

### 2.3. Gene Expression Analysis

Samples from the control and treated groups were collected for gene expression analysis at 24 and 48 h. The samples were prepared for total RNA assay using TRIzol (Invitrogen^®^, Carlsbad, CA, USA) according to the manufacturer’s instructions. The samples of *P. minimum* cells in TRIzol reagent were homogenized, followed by chloroform extraction and ethanol precipitation. Air-dried RNA pellets were dissolved in diethylpyrocarbonate-treated water. RNA was assayed by determining the absorbance readings at OD 260, and further assessed using 260/280 OD ratios and direct examination of the 28S and 18S bands on 1% agarose gels. Equal amounts of RNA were reverse transcribed into cDNA using MMLV Reverse Transcriptase (Invitrogen^®^, Carlsbad, CA, USA). The cDNA was used as a template for PCR amplification. Semi-quantitative reverse transcription PCR reactions were performed using a TaKaRa PCR Thermal Cycler system (Takara Bio^®^, Otsu, Japan) and AccuPower HotStart PCR PreMix (Bioneer^®^, Daejeon, Korea), according to the manufacturer’s instructions. Gene-specific primers used in this study are listed in Table 1.

The PCR conditions for all primers were as follows: first, primers were pre-denatured by heating to 95 °C for 10 min, followed by 30 cycles of denaturation at 95°C (30 s), annealing at 57 °C (10 s), elongation at 72 °C (30 s), and additional 10 min at 72 °C for the final elongation. After the reaction, the PCR mixture was electrophoresed on a 2% agarose gel and stained with ethidium bromide. Band intensities were quantified using image quant software (Multi Gauge V3.0, Fujifilm^®^, Tokyo, Japan). The relative changes in *Hsp*70, *Hsp*90, and *CYP* mRNA levels were normalized for Actin mRNA.

### 2.4. Statistical Analysis

The effects of tannic acid on the physiological parameters were statistically analyzed using one-way ANOVA at α = 0.05 (level of significance). IBM SPSS Statistics 22^®^ (New York, NY, USA) was used for the analysis.

## 3. Results

### 3.1. Physiological Response to Tannic Acid

**Figure 2:**
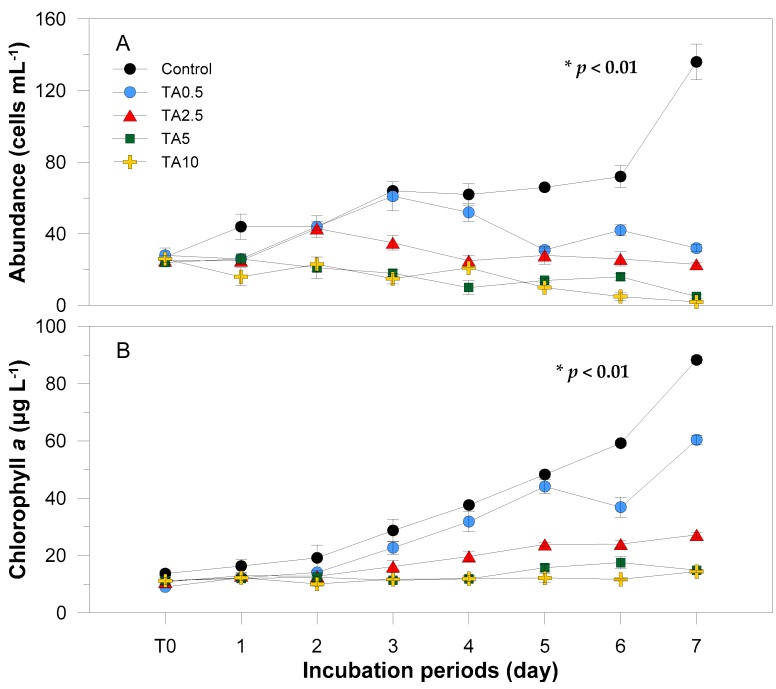
Variations in cell abundance (**A**) and chl *a* concentration (**B**) in the control and experimental groups to tannic acid (TA) during incubation periods. *: The effects of TA treatments were statistically significant at α = 0.05.

In the bioassay experiments, the initial population density of *Prorocentrum minimum* cultures at initial time (T0) were 26 ± 1.6 cells mL^−1^ (Figure 2A). The day after tannic acid was introduced, the cell count of the control group increased to 44 cells mL^−1^, while those of the experimental groups remained at levels similar to those observed at T0. On the second day, the cell densities of the low-concentration experimental groups (TA 0.5 and 2.5 ppm) were 44 and 43 cells mL^−1^, respectively, which is similar to that of the control group. The cell densities of the high TA concentration experimental groups (TA 5 and 10 ppm) were slightly lower than those at T0. During the third day, the cell densities of the control and TA-0.5 ppm group increased to 64 and 61 cells mL^−1^, respectively, while that of the 2.5 ppm group decreased to 35 cells mL^−1^. The cell densities in the TA 0.5 and 2.5 ppm groups tended to increase and decrease during the course of the incubation. There were also slight increments for the TA 5 and 10 ppm groups, but the final density at T7 showed almost no live cells (Figure 2B). The cell densities of all experimental groups at the end of the incubation either remained similar to the initial count or had decreased, whereas the control group showed a five-fold increase from its initial cell density.

The chl *a* initial concentration in all groups was 11.2 ± 1.7 μg·L^−1^. The control group continued to increase over the course of the experiment and was higher than that in the experimental groups at any given time. The groups in the 0.5 and 2.5 TA cultures, on the other hand, also increased but were not as high as the control. Meanwhile, the high concentrated TA groups demonstrated a more or less steady concentration. 

**Figure 3:**
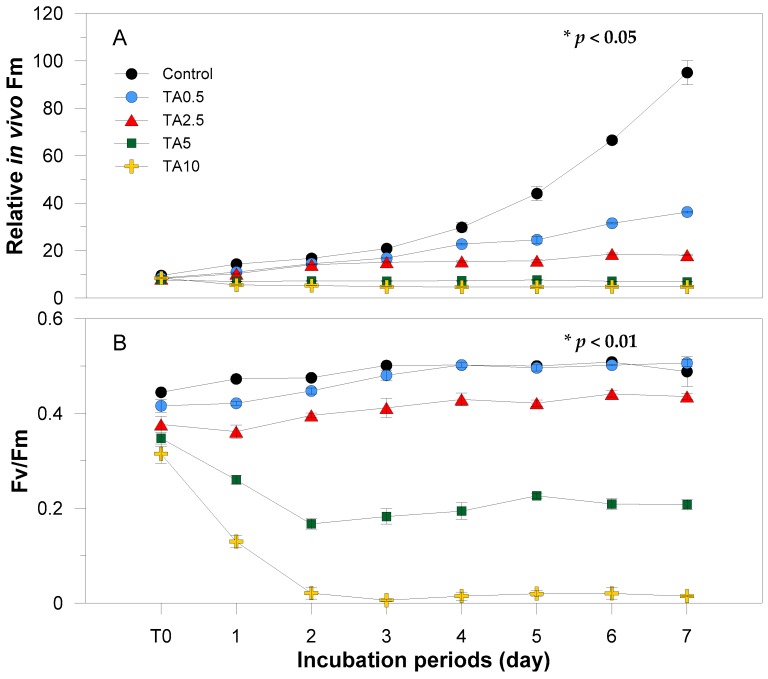
Variations in the *in vivo* Fm (**A**) and Fv/Fm (**B**) of the control and experimental groups to tannic acid (TA) during incubation periods. *: The effects of TA treatments were statistically significant at α = 0.05.

*In vivo* Fm followed a trend similar to that of chl *a* concentration (Figure 2B and Figure 3A). The initial value in all groups (experimental and control) was 8.4 ± 0.6. During the incubation period, the *in vivo* Fm in the control group continued to increase, showing a brighter fluorescence than the experimental groups. *In vivo* Fm was inversely proportional to the TA concentration gradient, as was the case with chl *a* concentration in the low-concentration groups. The TA 5 and 10 ppm groups showed very slight decreased Fm values compared to baseline fluorescence values (Figure 3A). 

Fv/Fm, representing photosynthetic efficiency, spontaneously reacted to the T0 concentration gradient of TA. The control group showed the highest Fv/Fm (mean: 0.49 ± 0.02; range: 0.44–0.51) throughout the incubation period. The Fv/Fm in the TA 0.5 and 2.5 ppm groups was 0.47 ± 0.04 (0.42–0.51) and 0.41 ± 0.03 (0.36–0.44), respectively, and that in the TA 5 and 10 ppm groups was 0.22 ± 0.06 (0.17–0.35) and 0.07 ± 0.11 (0.01–0.32), respectively, thus demonstrating sharp reductions during the first two days of incubation (Figure 3B).

The one-way ANOVA test confirms the significant differences (*p* < 0.05) in abundance, chl *a*, relative *in vivo* Fm, and Fv/Fm at various tannic acid concentrations (Figure 2 and Figure 3). 

### 3.2. mRNA Expression

**Figure 4:**
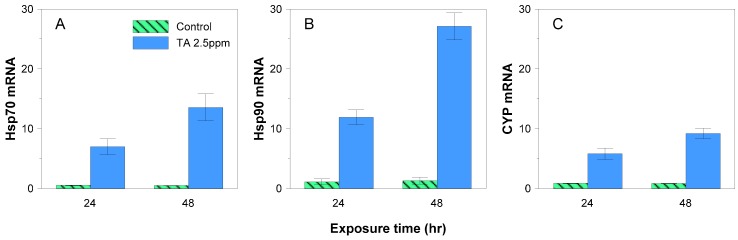
*Hsp*70 (**A**); *Hsp90* (**B**) and *CYP* (**C**) expression after 24 and 48 h of exposure to tannic acid (TA) at 2.5 ppm.

The TA-treated experimental groups showed sharp increments in the expression of *Hsp*70, *Hsp*90, and *CYP* mRNA compared to the levels in the control group, with the latter showing no change over the course of the experiment (Figure 4). By 48 h after TA treatment, *Hsp*70 and *Hsp*90 mRNA expression increased 20- and 27-fold compared to the control group. *CYP* mRNA expression showed a 10-fold increase by 48 h after TA treatment, thus exhibiting a relatively low rate of increase compared to the *Hsp*s. These results show that TA functions as a stress factor for *P. minimum*.

## 4. Discussion

### 4.1. Effects of Tannic Acid on Physiological Characteristics

**Figure 5:**
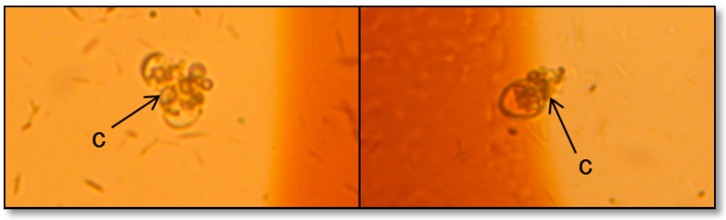
Chlorophyll pigments (**c**) released during cell rupture three days after treatment with tannic acid.

Tannic acid (TA) with its phenolic nature [50] behaves as a natural allelochemical in plants. It impedes phytoplankton growth [32] with various studies explaining its mode of inhibition. One study suggests that tannic acid inhibits the activity of critical proteins in cells [38,51] particularly alkaline phosphatase (APA) [52], which activates during low levels of inorganic phosphate. However, the media in this study was supplied with adequate phosphorus and the likely reason for *Prorocentrum minimum* growth inhibition could be the decline of photosystem II activity. Körner and Nicklisch [53] suggested this as the explanation for growth inhibition among other algal organisms particularly planktonic cyanobacteria, diatom, and green alga. This hypothesis is reflected in our results, wherein Fv/Fm responded immediately to tannic acid with measurable effects being visible even at T0 (Figure 3B). Set-ups with tannic acid concentrations of 5 and 10 ppm had a drastic inhibitory effect up until the second day of incubation and, by the third day, yield was more or less constant with 10 ppm set-up showing no photosynthetic activity. The consistency of the Fv/Fm starting from the second day up to the last day implies that the remaining cells have adapted to tannic acid concentration, but growth was stagnant. In order to fully eradicate the activity of *P. minimum,* a higher dosage of tannic acid should be applied. In the case of the low-concentrated experimental groups, on the second day of incubation, there was an increase in photosynthetic efficiency, and, on the fourth day, the group with 0.5 ppm tannic acid manifested the same efficiency level with the control group. The increasing yield of *P. minimum* treated with 0.5 and 2.5 ppm tannic acid is ascribable to the resistance and the adaptation of the species to low concentrations of tannic acid. This finding is further supported with both chl *a* concentrations and *in vivo* Fm gearing towards an increasing trend (Figure 2B and Figure 3A). Hypothetically, chlorophyll fluorescence usually follows a direct relationship with chlorophyll concentration within a linear range [54]. However, Ferreira and colleagues [55] argued that during *in situ* analysis, chl *a* fluorescence and concentration are not always directly proportional to each other. Other aspects such as temperature and high inorganic particle turbidity [55] can influence the variability of *in vivo* Fm. In the case of this study, the two parameters showed a positive relationship since measurement was done in cultured media where other factors are controlled. With regards to the increasing trend, a study by Herrera-Silveira and Ramirez-Ramirez [56] revealed that cultures exposed to lower concentrations of natural phenolic material, particularly tannic acid, have increased in phytoplankton biomass. They proposed that the interplay of other organic materials present in the medium has contributed to the increase in biomass. Furthermore, phytoplankton abundance decreases with time when treated with higher levels of phenolic substances. In this study, cells exposed to higher concentrations of tannic acid, chl *a* and *in vivo* Fm maintained a steady flow all throughout the incubation period. Microscopic examinations revealed that a large number of individuals have ruptured and chlorophyll leaked from the cells (Figure 5). The pigments remained in the cultured media, thus assuming to have been reflected in the chl *a* and *in vivo* Fm measurements.

During the last day of the incubation period, the groups treated with 0.5 and 2.5 ppm TA gave a similar cell density as that of their initial density although there were variations during the course of incubation (Figure 2A). On the other hand, those treated with 5 and 10 ppm tannic acid manifested a decline in abundance until almost none of the cells were viable at day 7. The abundance data somehow deviated from the trend of the aforementioned parameters. This result is not consistent with the findings of Herreira-Silveira and Ramirez-Ramirez [56], and the mechanism for the disagreement cannot be fully explained. This study suggests that microscope cell counting is not a robust method in measuring phytoplankton activity and that other factors should also be accounted. Photosynthetic efficiency (Fv/Fm) is a recommended parameter since it describes activity and can be in conjunction with phytoplankton count or components. 

Acid is known to enhance degradation of chlorophyll [57,58,59] even at a weak strength. One of the major byproducts of this degradation is pheophytin [60]. The structure of pheophytin is similar with chlorophyll except that the Mg^2+^ of the latter is replaced by H^+^. With the absence of the Mg^2+^ ion, the efficiency of photosystem II decreases since magnesium ions are involved in the absorption of the pigment array [61]; hence, photosynthetic efficiency also decreases. Another feature of tannic acid is its phenolic characteristic. The phenol groups attached to the main structure of tannic acid could be another reason as to why TA is capable of *P. minimum* growth decline. Phenols have the capacity to inhibit electron transport in photosystem II while the share of Q_B_-nonreducing centers is increased [62]. Meanwhile, Nakai *et al*. [63] suggest that phenols may auto-oxidize with di- or trivalent metal ions in the media causing the creation of radicals. These radicals pose toxicity to cells, eventually leading to apoptosis. Moreover, its allelopathic nature can cause cytolytic action such as perforating the cell membranes of phytoplankton organisms [64]. The free phenol forms of tannin can bind with carbohydrates and proteins which could be a reason for the lysis of *P. minimum* cells [65].

Enhancement of tannic acid toxicity is further aided by *in situ* conditions. Solar radiation is found to improve tannic acid allelopathy [66]. The presence of microbial entities could promote the sensitivity of phytoplankton species to tannic acid [67]. pH also significantly promotes tannic acid toxicity specifically in an alkaline environment [68]. Since saline water is basically alkaline in nature, application of tannic acid to *P. minimum* would be more effective. 

### 4.2. Effect of Tannic Acid on Gene Expression

The recent major trend in phytoplankton research is the investigation of its potential as a component of molecular technology. Genetic expression analysis is performed in order to detect various physiological responses at the molecular level. This study’s analysis of the stress-response genes (*Hsp*70, *Hsp*90, *CYP*) verified the increase in their expression under stress elucidating the physiological response of *P. minimum* to tannic acid. Heat shock proteins increase expression levels when exposed to stressors [69,70] especially those that denature proteins [71]. On the other hand, cyclophilins, being an integral part during PSII assembly and stabilization [72], upregulates under stress conditions such as oxidation, heat shock, salt stress, heavy metal toxicity, and chemicals [73,74,75]. In this paper, *Hsp* genes were more highly expressed than *CYP* genes (Figure 4), presumably because *Hsp* genes are more sensitive to chemical stress than *CYP* genes. In addition, between the *Hsp* genes, *Hsp*90 are more upregulated than *Hsp*70 in 48 h. 

The results of the gene expression analysis coincided with the physiological response of *P. minimum* to tannic acid. While the physiological parameter decreased in activity, the gene response elevated, signifying that starting at 2.5 ppm, tannic acid is considered stressful to the cells. Concentrations above 2.5 ppm may be beyond the capacity of the stress genes ultimately leading to the disruption of *P. minimum* physiology.

## 5. Conclusions 

This study utilized bioassays and molecular analyses to verify the efficacy of using tannic acid (TA) in inhibiting the growth of the noxious dinoflagellate, *Prorocentrum minimum* that blooms during the warm seasons in the Yeongsan River estuary, a eutrophic coastal system. Bioassay experiments and microscopic examination revealed that individuals exposed to low concentrations of TA (<2.5 ppm) have the ability to adapt and continue to grow, and exposure to high concentrations of TA would inhibit the development of *P. minimum*. Moreover, photosynthetic operation halted when 10 ppm was introduced to the cells. Tannic acid efficacy was further bolstered with the results of gene expression experiments, showing that the presence of the compound induces a stress response in *P. minimum*. A drastic increase in stress levels is likely to thwart metabolic processes and eventually inhibit growth over time. These findings confirm the applicability of the FRRf and gene-based validation methods in assessing physiological responses of phytoplankton. 

Tannic acid is a natural substance synthesized by common plants and is considered safe by the U.S. Food and Drug Administration [76]. Thus, TA treatment is a promising method for the control and mitigation of red tide organisms flourishing in coastal waters. Further research and validation will be necessary in order to test its ecological and environmental safety, impact on various organisms, and economic feasibility. This study is expected to serve as reference data for the development of technologies intended to control red tide organisms flourishing in eutrophic coastal waters.

## Figures and Tables

**Table 1:** Primers used in this study.

Gene Symbol	Nucleotide Sequence (5′→3′)	Length (bp)	Cycle	GenBank Access No. and Reference
Actin				
Forward	CAG CGG AAT TCA CGA CAC CAC C	117	25	JF715156.1
Reverse	CCG ATG CCT GGG AAC ATA GTC G			[47]
*Hsp*70				
Forward	TGA TCG GTC GCA AAT TCG CCG	120	30	JN401970.1
Reverse	TCT CCT CGC CCT GTG ATG TCA C			[45]
*Hsp*90				
Forward	ACG AGG ACT CCA CCA AC	120	30	JN831315.2
Reverse	TCT GGC CCT CCT TCA TAC GG			[46]
*CYP*				
Forward	AGT CCA TCT ACG GCA GCA AGT TTG	143	30	JF715159.1
Reverse	TCG AGC CAG GAA GTC TTC ACG G			[47]
